# Bone healing after median sternotomy: A comparison of two hemostatic devices

**DOI:** 10.1186/1749-8090-5-117

**Published:** 2010-11-24

**Authors:** Rikke F Vestergaard, Henrik Jensen, Stefan Vind-Kezunovic, Thomas Jakobsen, Kjeld Søballe, John M Hasenkam

**Affiliations:** 1 Dept. of Cardio-Thoracic and Vascular Surgery, Aarhus University Hospital, Skejby, Brendstrupgårdsvej 100, 8200 Aarhus N, Denmark; 2 The Institute of Clinical Medicine, Aarhus University Hospital, Skejby, Brendstrupgårdsvej 100, 8200 Aarhus N, Denmark; 3 The Orthopedic Research Laboratory, Aarhus University Hospital, Aarhus Sygehus, Aarhus C, Denmark

## Abstract

**Background:**

Bone wax is traditionally used as part of surgical procedures to prevent bleeding from exposed spongy bone. It is an effective hemostatic device which creates a physical barrier. Unfortunately it interferes with subsequent bone healing and increases the risk of infection in experimental studies. Recently, a water-soluble, synthetic, hemostatic compound (Ostene^®^) was introduced to serve the same purpose as bone wax without hampering bone healing. This study aims to compare sternal healing after application of either bone wax or Ostene^®^.

**Methods:**

Twenty-four pigs were randomized into one of three treatment groups: Ostene^®^, bone wax or no hemostatic treatment (control). Each animal was subjected to midline sternotomy. Either Ostene^® ^or bone wax was applied to the spongy bone surfaces until local hemostasis was ensured. The control group received no hemostatic treatment. The wound was left open for 60 min before closing to simulate conditions alike those of cardiac surgery. All sterni were harvested 6 weeks after intervention.

Bone density and the area of the bone defect were determined with peripheral quantitative CT-scanning; bone healing was displayed with plain X-ray and chronic inflammation was histologically assessed.

**Results:**

Both CT-scanning and plain X-ray disclosed that bone healing was significantly impaired in the bone wax group (p < 0.01) compared with the other two groups, and the former group had significantly more chronic inflammation (p < 0.01) than the two latter.

**Conclusion:**

Bone wax inhibits bone healing and induces chronic inflammation in a porcine model. Ostene^® ^treated animals displayed bone healing characteristics and inflammatory reactions similar to those of the control group without application of a hemostatic agent.

## Background

Cardiac surgery is predominantly performed through a median sternotomy. Today more than 700,000 sternotomies are performed each year in the USA alone [1]. This procedure provides excellent access to all mediastinal structures, is quick and easy to perform, and is well tolerated by most patients. Although complications are relatively rare, they are serious when they occur. Immediate complications are intra- and postoperative bleeding. These predispose to postoperative lack of bone healing which can lead to pseudoarthrosis and dehiscence or even infection and sternal erosion. To prevent bleeding, bone wax is traditionally used to physically block blood from oozing out of the spongy bone during operations which are performed during full heparinization. Bone wax consists of sterilized white-bleached honeybees wax (cera alba) blended with a softening agent, such as paraffin. The product is very effective for diminishing the amount of intraoperative bleeding. Bone wax unfortunately has significant potential long-term side effects. Thus, experimental studies have shown that when a bone defect is treated with bone wax, the number of bacteria needed to initiate an infection is reduced by a factor of 10,000 [2-4]. Furthermore, bone wax acts as a physical barrier which inhibits osteoblasts from reaching the bone defect and thus impair bone healing [5,6]. Once applied to the bone surface, bone wax is usually not resorbed [7].

Since intraoperative bleeding from the sternum can be excessive surgeons are often forced to balance the risk of blood loss against the long-term side effects of bone wax.

A new water-soluble polymer wax (Ostene^®^) has recently been introduced as a resorbable alternative to bone wax [8,9]. Ostene^® ^is used in the same way as bone wax to immediately ensure hemostasis by sticking to the bone surface and thus creating a physical barrier. The biocompatible polymers used in Ostene^® ^have been shown to be eliminated from the body and remain unchanged through renal clearance [10]. The properties of Ostene^® ^are claimed to mimic the ideal hemostatic properties of bone wax while avoiding the inherent risks of infection and impaired bone healing associated with the use of traditional bone wax. Based on this we hypothesized that Ostene^® ^would have a lesser impairing effect on bone healing and lead to a reduced inflammatory response compared to bone wax. Accordingly we aimed to compare bone healing and inflammation in three groups of pigs receiving either bone wax, Ostene^®^, or no local hemostatic treatment as an adjacent procedure to sternotomy.

## Materials and methods

All animal handling and caretaking was conducted in accordance with guidelines given by the Danish Inspectorate of Animal Experimentation and after approval from this institution.

**Figure 1:**
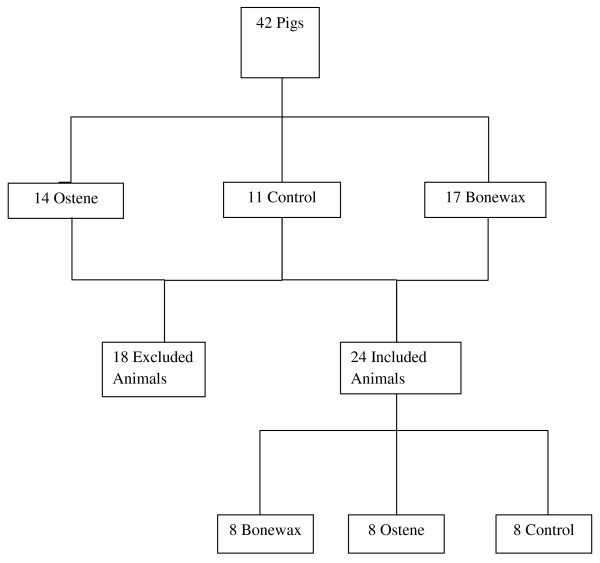
**Flowchart: showing how the pigs were in- and excluded**.

Among 42 Danish landrace female pigs with a mean body weight of 50 kg, 24 were included in the study. The 18 remaining pigs were excluded because of deep sternal infection, death during surgery, or euthanasia due to poor thriving before scheduled termination (Figure 1 and Table 1).

**Table 1:** Exclusion of animals: Distribution of the excluded animals

Exclusion Criteria	Bone wax-group	Ostene^®^-group	Control-group
Sternal infection	2	3	3

Intraoperative death	2	1	0

Poor Thriving	4	2	0

Non-Union	1	0	0

### Surgical procedure and postoperative care

After induction of general anesthesia each animal was randomized into one of three treatment groups: Ostene^®^, bone wax, and a control group receiving no hemostatic treatment. The animals were then subjected to a midline sternotomy with an oscillating saw. Standard aseptic surgical techniques were used. In the first two groups, either Ostene^® ^(supplied by Ceremed, Inc., 3643 Lenawee Avenue, Los Angeles, California, USA) or bone wax (Braun Aesculap AG & CO. KG) was applied to both spongiosa surfaces until bleeding had ceased.

Electro cauterization was used on the superficial and profound surfaces of the sternum in all three groups. The sternotomy was left open for 60 min before closure commenced to simulate conditions similar to those in standard cardiac operations. The sternum was then closed using rigid osteosynthesis by a compression screw through the two cranial costae and 12 single steel wire sutures (Monofilament 316L Stainless steel non-absorbable sutures, Syneture, Covidien, 15 Hampshire Street, Mansfield, MA, USA). Subcutaneous and skin tissues were closed in three layers (for the fasciae and muscle layers: 0 Polysorb, Syneture,. For the intradermal sutures: 3-0 Biosyn, Syneture,. For the skin: 0 Surgipro, Syneture,). The skin sutures were removed after ten days.

All animals received the same pre-and postsurgical medication:

• Antibiotics in terms of cephalosporins(1500 mg) before and after the surgery and for three days post surgically and locally applied ampicillin during surgery.

• Pain-reducing regimen with NSAID (250 mg), morphine (100 μg/hour) and opioids (0.15 mg) after the surgery and for three days post surgically.

The animals were returned to the farming facilities on the day of surgery for postoperative care for six weeks. Care was performed by qualified animal caretakers. The animals were then euthanized with a captive bolt pistol and the sternum was removed.

### Specimen preparation

The sternal body was separated from the manubrium at the manubriosternal-joint surface and the xiphoid process was removed. From each sternal body one sample with a length of two cm was cut for histological analysis from the caudal part of the sternum and the rest was immediately frozen at -18°C. Preparation of specimens and subsequent evaluation were conducted in a blinded fashion.

### Analysis

#### Peripheral Quantitative Computerized Tomography (PQ-CT)

The bone density in the center of the frozen bone was measured by peripheral quantitative Computerized Tomography (PQ-CT), using an XCT 2000 scanner from Stratec Biomedical Systems AG (Gewerbestr. 37, 75217 Birkenfeld, Germany). PQ-CT is a method of accessing bone mineral density which uses multiple cross-sectional x-ray images to reconstruct a volumetric model of the bone density distribution. The analyzed bone mineral density is presented as mg/cm^3^.

**Figure 2:**
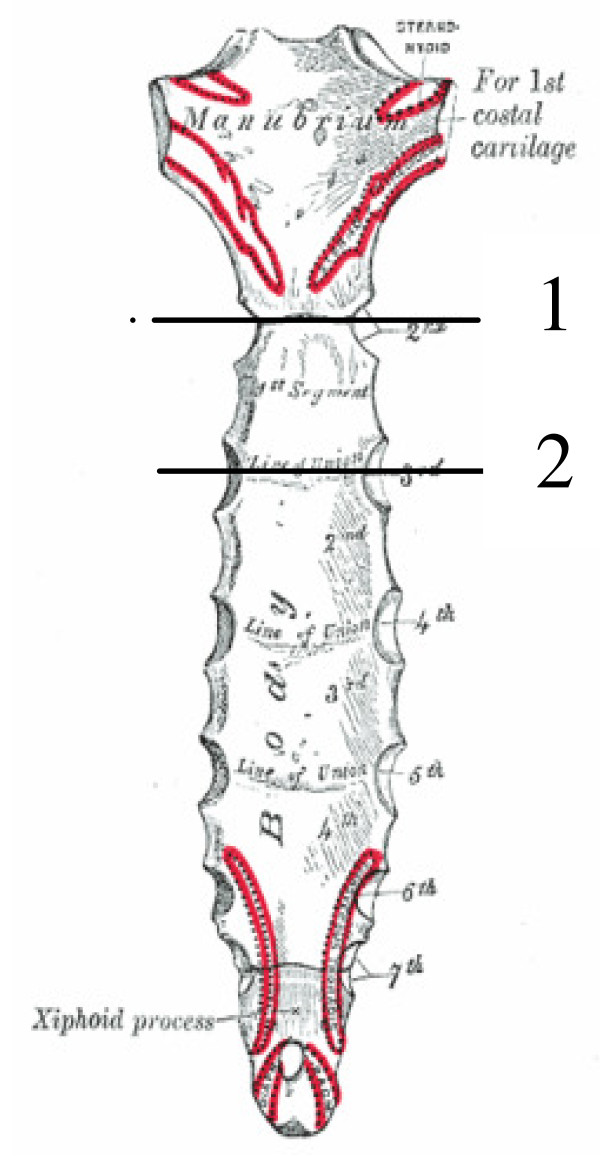
**CT reference lines: Sternum showing the two reference-lines used in the CT-scans**. 1: Manubriosternal joint surface. 2: First growth-zone

**Figure 3:**
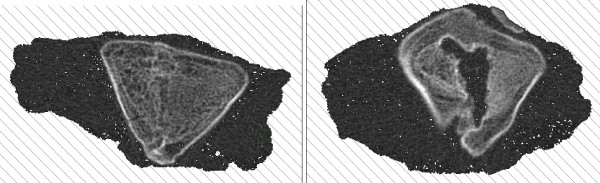
**CT-images: CT-images showing good central healing of the sternum in a control pig (left) and decreased central healing in a bone wax pig (right)**.

The manubriosternal-joint surface was used as one reference point and the first growth zone was used as a second reference point (Figure 2). Three images 0.3 mm apart were made 10 mm caudal from each reference point. On each image a region of interest (ROI) with an area of approximately 20 mm^2 ^was identified. The ROI was located in the least dense part of the bone (determined visually) and in such a way that it included only trabecular bone and no cortical bone (Figure 3). Subsequently the total area of the defect was determined.

#### Histology

One block of 2 cm length was cut from the caudal end of the sternal body and gradually dehydrated in ethanol (70-100%) and embedded in methylmethacrylate (MMA) and then sectioned. Four sections separated by 500 μm were cut from the block using a hard tissue microtome (KDG-95, MeProTech, Heerhugowaard, The Netherlands) and from each level five slices of 7 μm thickness were cut and stained with Goldners Trichrom, which stains mineralized bone green and non-mineralized bone red.

The sections were cut in the anterior-posterior direction so they represent the entire cross-section of the sternum.

A stereological software program (CAST-grid Olympus Denmark A/S, Ballerup, Denmark) was used for this analysis. Fields of vision from a light microscope were displayed on a computer screen at 4× magnification. A user-specified point grid with 24 crosses was superimposed onto the microscopic fields, and the sampling-technique used was meander sampling with a step length of 2500 μm. A random representative 24% of the tissue on the slice was counted. Any granuloma that transected the upper right quadrant of a cross was counted. A granuloma was defined as an aggregate of epitheloid histiocytes and foreign body giant cells surrounded by fibrotic tissue. Total counted tissue is defined as the sum of all counted tissue types (bone marrow, mineralized bone, unmineralized bone, fibrotic tissue, cartilage, muscle fibers and fatty tissue and granulomas).

The ratio of granulomas was calculated using this formula:

Ratio of granuloma=counted granuloma/total counted tissue

#### X-ray

Three categories of healing were visually determined by measuring the gap between the bone surfaces 1:1 scale X-ray images:

1. Total bone healing (perfect alignment of the bone surfaces with no discernable gap)

2. Partial bone healing (misalignment of the bone surfaces with a gap of 5 mm or less)

3. No healing (gap greater than 5 mm)

### Statistical handling

Data was checked for normal distribution. Students T-test was applied on PQ-CT density-data to test for differences between treatment groups. Mann-Whitney rank sum test was used for the X-ray- and histology data as well as the area of the central defect. P values of less than 0.05 were considered statistically significant.

## Results

**Figure 4:**
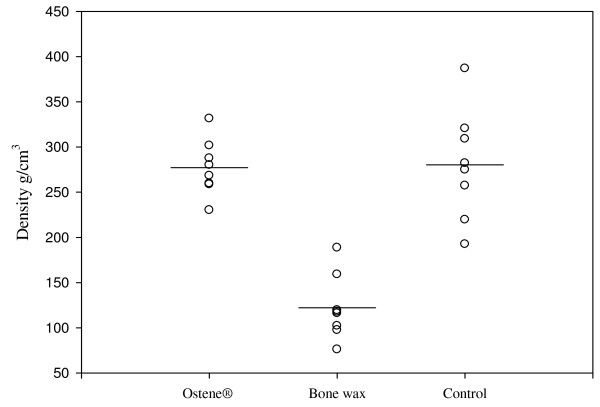
**CT-results showing the bone-density measured in g/cm^3^: The difference between bone wax and Ostene^® ^and bone wax and control = p < 0.001**. Means are indicated by a vertical line.

**Figure 5:**
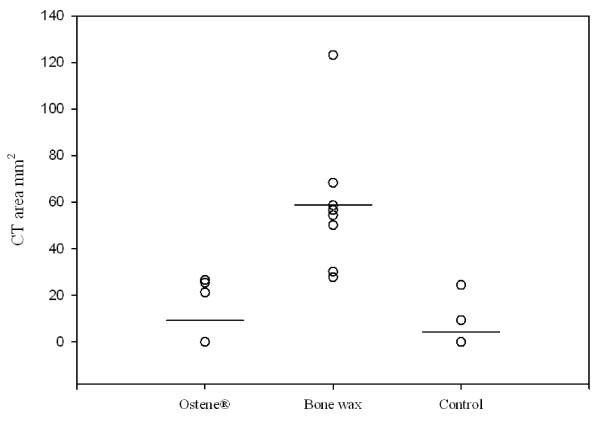
**CT-results showing the area of the central defect in the first sternal segment: The difference between bone wax and Ostene^® ^and bone wax and control = p < 0.001**. Means are indicated by a vertical line.

**Figure 6:**
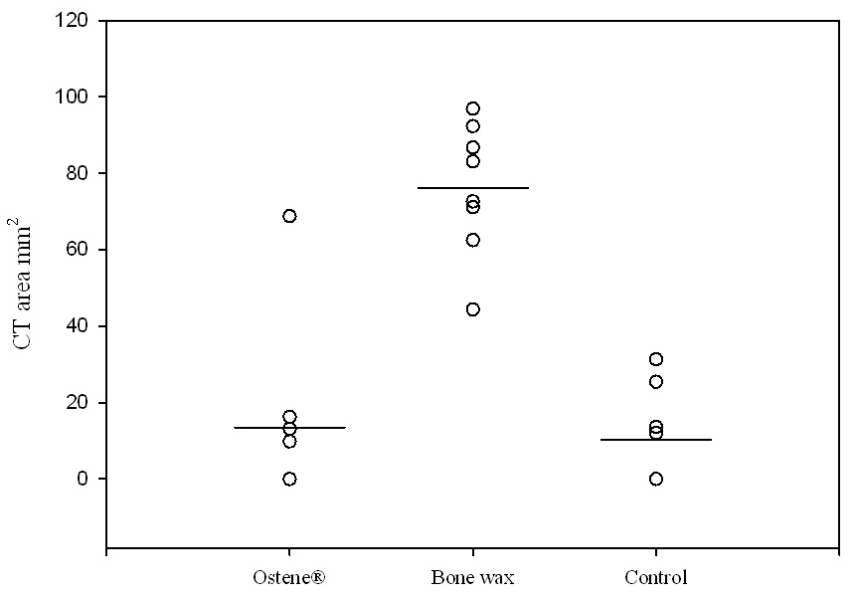
**CT-results showing the area of the central defect in the second sternal segment**. The difference between bone wax and Ostene^® ^and bone wax and control = p < 0.001. Means are indicated by a vertical line.

Deep sternal wound infections were distributed evenly across the groups and these animals were not included for further analysis (Table 1) PQ-CT revealed that the sternum of pigs treated with bone wax had a significantly lower bone density and the area of the central defect was significantly higher compared with both the control and Ostene^® ^groups (p <0.001) (Figure 4, 5 and 6). There was no significant difference between the two latter groups. These findings were supported by the X-ray analysis (Table 2), which also showed that there was significantly less healing in the bone wax groups compared with both the control and Ostene^® ^groups. Again, no significant difference between the two latter groups could be found.

**Table 2:** X-ray result: The table shows how the images were allocated according to group and the statistical difference between them.

Ostene^®^		Bone Wax		Control	
Total Healing	2	Partial Healing	1	Total Healing	2
Partial Healing	1	No Healing	0	Total Healing	2
Partial Healing	1	Total Healing	2	Total Healing	2
Total Healing	2	No Healing	0	Partial Healing	1
Total Healing	2	Partial Healing	1	Total Healing	2
Partial Healing	1	Partial Healing	1	Total Healing	2
Total Healing	2	Partial Healing	1	Total Healing	2
Total Healing	2	No Healing	0	Total Healing	2
Mean	1.6		0.8		1.9
Sd	0.5		0.7		0.4

					
				
				p-value	
		
		Ostene™ vs. Bone wax		0.02	
		Ostene™ vs. Control		0.26	
		Bone wax vs. Control		0.0035	

The difference between bone wax and Ostene^® ^and bone wax and control = p < 0.001.

1. Total bone healing (perfect alignment of the bone surfaces with no discernable gap)

2. Partial bone healing (misalignment of the bone surfaces with a gap of 5 mm or less)

3. No healing (gap greater than 5 mm)

**Figure 7:**
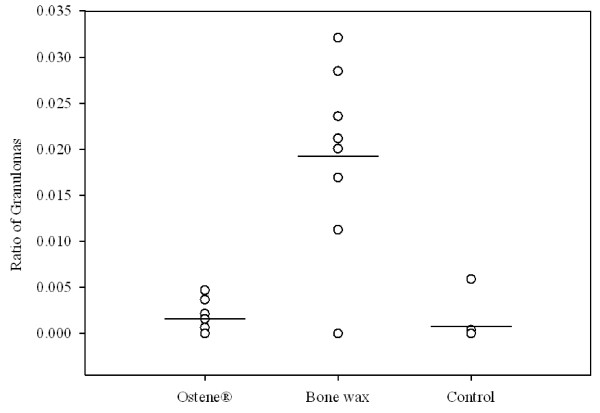
**Histology results showing the volume fraction of granuloma to other tissue: The difference between bone wax and Ostene^® ^and bone wax and control = p < 0.001**. Means are indicated by a vertical line.

Histology results revealed a significantly larger ratio of chronic inflammation, granulomas, in the bone wax group compared with the control (p = 0.003) and Ostene^® ^groups (p = 0.007) (Figure 7). There was no significant difference between the two latter groups.

## Discussion

Due to aggressive pre- and post surgical antibiotic regiments and modern wound management the rate of sternal wound infection and dehiscence has been greatly reduced to approximately 1-2%. But patients with much co-morbidity are still faced with a high risk, up to 14.3%, for these complications [11], which are associated with an increased mortality, up to 47% [12]. Therefore, a search for ways to prevent these complications is still warranted.

Our study shows that bone wax leads to chronic inflammation and reduced bone healing were as Ostene^® ^does not and there are no significant differences in bone healing when comparing Ostene^® ^to no hemostatic treatment.

Several previous experimental studies have shown that bone wax inhibits bone healing and induces inflammation [3-5,10]. Similar findings in human studies have been reported but mostly as case reports and retrospective studies [2,6,13]. A larger controlled randomized study was recently published, comparing bone wax to a control group receiving no hemostatic treatment with regards to sternal infection among other things. No link between sternal infection and bone wax could be shown, but there was a very low incidence of infection both groups, suggesting that the results may be due to a lack of power [14]. It would be quite difficult to show a link between bone wax and sternal wound infection in a cardiac surgery population as the incidence generally is between 1 and 2%, so a very high number of subjects would be necessary in both the bone wax and the control group to show any statistically significant results [15].

Our study has certain limitations. Firstly, the results might just reflect delayed bone healing in the pigs treated with bone wax, and it is possible that these might catch up to the pigs treated with Ostene^® ^at some later time point. However, since the control animals depicted total sternal healing after 6 weeks no doubts can be raised that bone wax significantly disrupted sternal healing in the immediate period following surgery and in this period sternal stability is crucial to avoid sternal nonunion and possibly infection [16-18].

Secondly, the reduced bone density does not necessarily predict the sternal stability or strength of the bone. It would be of interest to examine the mechanical strength of the bone.

Other surgical specialties have far more restrictive policies regarding the use of bone wax. For instance in neurosurgery and oral surgery the use of bone wax has been linked to surgical site infection as well as foreign body granuloma and nerve damage [2,6,19,20].

Effective hemostatic treatment is of paramount importance in any surgical setting, but the drawbacks of bone wax must lead to careful consideration by surgeons before use. Ostene^® ^presents an effective alternative to bone wax. Neither of the substances has any inherent blood-clotting properties. They act purely by forming a mechanical barrier which prevents the flow of blood oozing from the exposed spongy bone and thus induces hemostasis.

It would be preferable to use no hemostatic agents at all during surgery, but this is not always a viable option since postoperative bleeding can lead to hematomaformation constituting a risk of infection in itself. Thus Ostene^® ^presents a potentially less risky alternative for open heart surgery and other surgical situations where the use of hemostatic devices is necessary to achieve better overlook of the surgical field and reduce the amount of intraoperative bleeding.

## Conclusion

Our results show that bone wax significantly inhibits bone-healing and induces chronic inflammation in pigs whereas Ostene^® ^does not. These results indicate that the use of this product instead of bone wax could contribute to a reduction in the incidence of sternal dehiscence and chronic inflammation.

## Competing interests

The manufacturer of Ostene^® ^contributed with partial financial support for this study, but no salary was received. We have reserved the right to publish our result regardless of the findings.

None of the authors have any financial ties to Ceremed or hold in stocks or shares in this enterprise.

None of the authors have non-financial ties to Ceremed.

## Authors' contributions

JMH and KS were both involved in the conception of the study and the study design as well as drafting and revising the article. HJ and SV-K both contributed to the surgical procedures and the acquisition of data as well as the data analysis. TJ contributed to data acquisition and analysis. RFV was involved in all the above mentioned study parts. All authors have approved the manuscript.
